# Comment on: Correlation between acetabular index at 3 and 12 months of age: a longitudinal radiographic study of 228 neonates treated for 6 or 12 weeks with the von Rosen splint for developmental dysplasia of the hip

**DOI:** 10.2340/17453674.2026.45783

**Published:** 2026-04-16

**Authors:** Soner KOCAK

**Affiliations:** University of Health Sciences, Istanbul Kanuni Sultan Suleyman Training and Research Hospital, Department of Orthopaedics and Traumatology, Istanbul, Turkey

*Sir,* — I read with great interest the longitudinal study by Sand et al. examining the correlation between the acetabular index (AI) at 3 and 12 months in neonates treated with the von Rosen splint for developmental dysplasia of the hip (DDH) [1]. Their central finding—namely, that the correlation was only moderate and that individual changes in AI varied widely—is clinically important because it challenges the common assumption that an early radiographic acetabular angle can reliably forecast later acetabular development. In my view, this is the main contribution of the article and one that deserves emphasis.

The study also raises a useful caution regarding the interpretation of very early post-treatment imaging. The authors show that mean AI trajectories differed between the 6-week and 12-week treatment groups, yet these groups were derived from different hospitals and different treatment eras. As a result, the observed differences at 3 months may not be attributable solely to treatment duration. This does not diminish the value of the report; rather, it highlights how sensitive early AI may be to contextual factors such as timing of imaging, local treatment routines, and cohort composition. For that reason, the conclusion that 3-month AI should not be used in isolation to trigger additional abduction treatment appears both reasonable and clinically prudent.

At the same time, the article opens an important methodological discussion. Hip-level analyses that include both pathological and contralateral “healthy” hips may increase statistical power, but they can also blur the distinction between prediction and association, particularly when measurements from 2 hips within the same child are not fully independent. Moreover, correlation coefficients describe linear association rather than clinical usefulness. A modest r value may coexist with acceptable discrimination in selected subgroups, whereas a statistically significant correlation may still be unhelpful for treatment decisions. Future analyses would therefore be strengthened by patient-level or mixed-effects models, together with threshold-based outcomes that matter to clinicians, such as persistent dysplasia at 18 to 24 months, need for prolonged surveillance, or later corrective surgery.

This is where the present work can make a broader contribution. Rather than asking whether AI at 3 months is predictive on its own, a more clinically relevant question may be whether early AI adds incremental value to a composite risk model. Recent follow-up studies after brace treatment suggest that residual acetabular dysplasia is better understood through longitudinal risk stratification than through a single early measurement: persistent dysplasia at 2 years may identify the children who require continued follow-up, whereas children who are radiographically normal at 2 years appear unlikely to show later deterioration [2,3]. In this context, a 3-month radiograph may remain highly valuable for detecting treatment failure or persistent dislocation, but less suitable as a standalone decision tool for escalation of treatment.

A logical next step would be a prospective multicenter study combining early radiographic variables with dynamic or static ultrasound features, laterality, treatment initiation age, and subsequent imaging at 12, 18, and 24 months. Such a design could determine whether a combined model—rather than AI alone—identifies a subgroup at genuine risk of residual dysplasia. This would move the field from descriptive association toward actionable prediction and could help reduce both unnecessary follow-up and missed persistent dysplasia.

Sand et al. should be commended for questioning an entrenched assumption in DDH surveillance. Their findings support continued use of the 3-month radiograph to identify rare early failures, but they also invite a more nuanced, risk-adapted framework in which later radiographic normalization and multimodal imaging features, rather than early AI alone, guide follow-up intensity.


**Soner KOCAK** *University of Health Sciences, Istanbul Kanuni Sultan Suleyman Training and Research Hospital, Department of Orthopaedics and Traumatology, Istanbul, Turkey* *Correspondence: dr.sonerkocak@gmail.com*


## References

[1] Sand A, Wenger D, Düppe H, Tiderius C J. Correlation between acetabular index at 3 and 12 months of age: a longitudinal radiographic study of 228 neonates treated for 6 or 12 weeks with the von Rosen splint for developmental dysplasia of the hip. Acta Orthop 2026; 97: 9-13. doi: 10.2340/17453674.2025.45043. 41482931 PMC12766171

[2] Saeed A, Bradley C S, Verma Y, Kelley S P. Resolving residual acetabular dysplasia following successful brace treatment for developmental dysplasia of the hip in infants. Bone Joint J 2024; 106-B(7): 744-50. doi: 10.1302/0301-620X.106B7.BJJ-2023-1169.R1. 38945534

[3] Larwood J P J, Idowu O, Elliott K G B, Lindisfarne E A, Aarvold A. Follow-up after successful treatment for developmental dysplasia of the hip using a Pavlik harness: is two years enough? Bone Joint J 2025; 107-B(4): 495-500. doi: 10.1302/0301-620X.107B4.BJJ-2023-1213.R2. 40164190

